# Effects of Gut Metabolites and Microbiota in Healthy and Marginal Livers Submitted to Surgery

**DOI:** 10.3390/ijms22010044

**Published:** 2020-12-22

**Authors:** Marc Micó-Carnero, Carlos Rojano-Alfonso, Ana Isabel Álvarez-Mercado, Jordi Gracia-Sancho, Araní Casillas-Ramírez, Carmen Peralta

**Affiliations:** 1 Institut d’Investigacions Biomèdiques August Pi i Sunyer (IDIBAPS), 08036 Barcelona, Spain; mico@clinic.cat (M.M.-C.); rojano@clinic.cat (C.R.-A.); 2 Departamento de Bioquímica y Biología Molecular II, Escuela de Farmacia, Universidad de Granada, 18071 Granada, Spain; analvarezmercado@gmail.com; 3 Institut of Nutrition and Food Technology “José Mataix”, Center of Biomedical Research, University of Granada, 18016 Granada, Spain; 4 Instituto de Investigación Biosanitaria ibs, GRANADA, Complejo Hospitalario Universitario de Granada, 18014 Granada, Spain; 5 Liver Vascular Biology Research Group, Barcelona Hepatic Hemodynamic Laboratory IDIBAPS, 03036 Barcelona, Spain; Jordi.gracia@idibaps.org; 6 Centro de Investigación Biomédica en Red de Enfermedades Hepáticas y Digestivas (CIBERehd), 08036 Barcelona, Spain; 7 Hospital Regional de Alta Especialidad de Ciudad Victoria “Bicentenario 2010”, Ciudad Victoria 87087, Mexico; aranyc@yahoo.com; 8 Facultad de Medicina e Ingeniería en Sistemas Computacionales de Matamoros, Universidad Autónoma de Tamaulipas, Matamoros 87300, Mexico

**Keywords:** microbiota, liver transplantation, partial hepatectomy, liver surgery, ischemia-reperfusion

## Abstract

Microbiota is defined as the collection of microorganisms within the gastrointestinal ecosystem. These microbes are strongly implicated in the stimulation of immune responses. An unbalanced microbiota, termed dysbiosis, is related to the development of several liver diseases. The bidirectional relationship between the gut, its microbiota and the liver is referred to as the gut–liver axis. The translocation of bacterial products from the intestine to the liver induces inflammation in different cell types such as Kupffer cells, and a fibrotic response in hepatic stellate cells, resulting in deleterious effects on hepatocytes. Moreover, ischemia-reperfusion injury, a consequence of liver surgery, alters the microbiota profile, affecting inflammation, the immune response and even liver regeneration. Microbiota also seems to play an important role in post-operative outcomes (i.e., liver transplantation or liver resection). Nonetheless, studies to determine changes in the gut microbial populations produced during and after surgery, and affecting liver function and regeneration are scarce. In the present review we analyze and discuss the preclinical and clinical studies reported in the literature focused on the evaluation of alterations in microbiota and its products as well as their effects on post-operative outcomes in hepatic surgery.

## 1. Introduction

Liver transplantation (LT) faces an urgent problem due to the shortage of liver grafts available for transplant. With the aim of resolving this problem, the criteria for discarding liver grafts have been changed. Thus, organs with diseases such as steatosis and positive hepatitis B or C have been used in LT [1]. Liver steatosis is a key factor when evaluating donor livers because of the high prevalence (30% in cadaveric and 20% in living donors) of negatively affecting recipient outcomes [1]. Thus, non-alcoholic fatty liver disease (NAFLD) is a common cause of liver rejection [2]. However, it is known that these types of livers are less functional and more sensitive to the injurious effects induced by ischemia-reperfusion (I/R), which is associated with graft dysfunction or primary non-function after transplantation [1,3]. Also, it should be noted that I/R negatively affects the regenerative capacity of the liver after a partial hepatectomy (PH), which might result in liver failure and poor post-operative outcomes [4].

In LT and PH surgery, the damage induced by I/R (which is exacerbated by diseases such as steatosis or cirrhosis) originates from the loss of blood supply during ischemia and its reestablishment during the reperfusion phase. This initiates a cascade of pathological features leading to an increase of reactive oxygen species (ROS), cytokines and neutrophil accumulation, resulting in inflammation, regenerative failure and cell death [5]. Since I/R is an inherent part of the surgical process in hepatic resections and LT [6], the numerous studies reported in the literature have mainly focused on procedures directed at the liver itself to reduce the injurious effects of ROS through the administration of antioxidants [7], to reduce neutrophil accumulation through treatment with antibodies anti-P-selectin or anti-intercellular adhesion molecule 1 (anti-ICAM) [8,9] or to regulate the activity or levels of some cytokines involved in the inflammatory process, such as tumor necrosis factor (TNF) or interleukin-6 (IL-6) [10]. However, the hepatic I/R associated with hepatic resections and LT (especially in the presence of liver disease) remains an unresolved problem in clinical practice. In our view, alternative strategies that are not focused exclusively on the liver, and studies to evaluate options other than the liver as the main target for reducing the mechanisms responsible for the pathologies associated with hepatic resection and LT are required.

### Relevance of the Gut–Liver Axis

Given the observations mentioned above, the current review will focus on the gut–liver axis (a consequence of the close anatomical and functional bidirectional interaction between the gastrointestinal tract and liver, primarily through the portal circulation) [11] in hepatic surgery, and will investigate the potential existence of a relationship between liver surgery and changes in the gut microbiota [12,13]. Indeed, some studies suggest that alterations in the gut microbiota might be responsible for the post-operative outcomes in different pathologies, which require the presence of a relationship between the intestine and the liver. Such is the case with the clinical surgical procedures of hepatic resections and LT [14,15].

The gut microbiota (GM) is a microbial community living in symbiosis both between constituents and with the human. The majority of its species are commensals, mainly from the Firmicutes and Bacteroidetes phyla, but there are also other phyla such as Proteobacteria or Actinobacteria [16,17].

Currently, many findings have shown alterations to GM in pathological conditions such as cardiovascular disease [18,19], cirrhosis [20], insulin resistance [21,22] or inflammatory bowel disease [23]. These alterations in GM might negatively affect recovery time and quality in patients, in addition to the effects of the different treatments applied. To elucidate the relevance of GM, investigations aimed at evaluating the signaling pathways involved in the gut–liver axis have been performed [4,17,24]. If there is dysbiosis, the microbiota is not properly balanced, thereby inducing an increase in intestinal permeability. If the intestinal barrier is affected, products present in the gut can reach the liver, including some that are toxic to the organism resulting in hepatic inflammation and the consequent development from simple steatosis to non-alcoholic steatohepatitis (NASH) [25,26]. This progression has been related with cytotoxicity resulting from the increases in fecal bile acids (BAs) and primary/secondary BA ratio, plasma and liver BA levels and plasma lipopolysaccharides (LPS) [25,27,28]. NAFLD patients showed fewer amounts of *Bacteroidetes, Ruminococcaceae* and *Faecalibacterium prausnitzii* and greater amounts of *Prevotella, Porphyromas, Lactobacillus, Escherichia, and Streptococcus* than healthy subjects [26,29].

In cirrhotic animals, an increase in the *Firmicutes* and *Actinobacteria* compared with control mice has been described [30]. However, increased levels of *Veillonella, Megasphaera, Dialister, Atopobium,* and *Prevotella* has been observed in cirrhotic patients [31]. Preclinical results indicated that HFD diet and intestinal Gram-negative bacteria resulted in liver fibrogenesis [32]. However, LPS reduction and intestinal tight junctions (TJs) restoration might be a therapeutic strategy for the treatment of fibrosis in NASH [33].

The gradual alcoholic liver disease (ALD) at early disease stages is related to dysbiosis and increased microbial translocation. The bacterial species related with such changes include *Streptococcus, Shuttleworthia,* and *Rothia* [34]. In long-time alcohol consumers, it has been described as a rise of Gram-negative bacteria, which cause endotoxemia and hyper-activation in the immune system [35]. The diminution in the *Roseburia* abundance is related to alcohol consumption in the human cohort [36], wheras *Roseburia* administration in an experimental ALD model improves hepatic steatosis and inflammation [36].

Chen et al. [37] describes genetic and microbial associations to plasma and fecal BA concentrations and composition in obese patients and establish their relationships with liver fat. The authors reported several microbial species that clustered together and showed strong positive correlations with secondary BA and negative correlations with primary BA entities. These included microbial species capable of mediating the conversion of primary BAs into secondary ones such as *Eubacterium hallii* [38] *or Ruminococcus torques* [39] and *Ruminococcus sp_5_1_39BFAA* [40], positively correlated with the levels of secondary/primary BA ratio in plasma. The study reported by Kurilshikov et al. [40] presents the largest metagenome-based association on plasma metabolism and microbiome relevance to diet, inflammation and cardiovascular disease (CVD) risk in obese patients. The authors indicated that *Ruminococcus_5_1_39BFAA* were associated to liver fat content in obese patients. Moreover, the amount of the anti-NAFLD species *Faecalibacterium prausnitzii* [41] is negatively correlated with multiple BA entities and hepatic fatty infiltration as well as intestinal and adipose tissue inflammation.

Given all the data mentioned above, alterations in gut metabolites and microbiota are involved in the pathogenesis and progression of NAFLD. In addition, gut microbial composition and function varies between individuals in different liver diseases.

**Figure 1:**
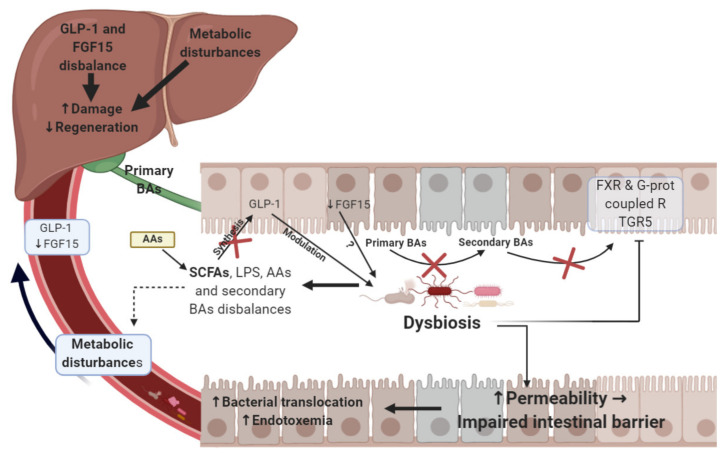
Association between gut microbiota and liver function in liver surgery. Gut dysbiosis leads to an imbalance in microbial metabolites such as SCFAs, LPS or secondary BAs, thus provoking metabolic disturbances and affecting GLP-1 and FGF15 synthesis or FXR and G-protein coupled receptor TGR5 signaling. All of this may increase liver damage and decrease liver regeneration ability. Dysbiosis also seems to alter gut permeability, inducing damaging effects on the intestinal barrier and causing bacterial translocation and endotoxemia. Gut bacteria also provide amino acids to the host and can alter their bioavailability. In addition, amino acids act as precursors for the synthesis of metabolic end products produced by the microbiota such as SCFAs. ↑: Increase; ↓: Decrease; AAs: Amino acids; BAs: Bile acids; FXR: Farnesoid X receptor; FGF15: Fibroblast growth factor 15; GLP-1: Glucagon-like peptide 1; LPS: Lipopolysaccharides; R TGR5: Receptor of Takeda G protein-coupled receptor 5; SCFAs: Short-chain fatty acids.

The current review will analyze and discuss the preclinical and clinical data reported in the literature about potential alterations in the microbiota and its products, and their effect on post-operative outcomes in different types of liver submitted to either hepatic resection or LT. Whether the intestinal dysbiosis or alterations in products derived from the microbiota are a cause or a consequence of liver damage in surgical conditions is of both scientific and clinical interest [24] and will also be discussed. This is because the liver might receive products derived from the gut microbiota, such as short-chain fatty acids (SCFAs), LPS, secondary BAs and amino acids (AAs). The regulation of these products, in which the liver is involved, is important to keep an organism healthy [12]. On the other hand, alterations in such products might negatively affect the quality of transplanted livers and post-operative outcomes (Figure 1) [42]. The mechanisms involved in alterations of the gut microbiota, as well as regulation of the same by different treatments will also be evaluated and discussed. This could contribute to the design of appropriate preclinical models of surgery and the establishment of new strategies that might be useful in the clinical practice of hepatic resections and LT.

## 2. Relationship between Liver Transplantation and Gut Microbiota

Numerous studies have demonstrated that gut dysbiosis is one of the main contributors to end-stage liver disease progression, associated with its severity, and morbidity and mortality rates [17,43,44]. Nevertheless, its role in patients following LT remains poorly understood.

The microbiota seems to play an important role in post-operative outcomes according to data reported by many authors [45]. However, it has been suggested that post-surgery prognosis depends on multiple factors such as ischemia-reperfusion injury [46], immunosuppressive drugs [47] or the appropriateness of matching based on donor-*organ*-recipient variables [48].

The gut microbiota acts by metabolizing bile salts with the aim of neutralizing their toxicity, transforming primary bile acids into secondary ones, thereby modifying the size and composition of the bile acid pool secreted by the liver [49]. BAs contribute to the activation of nuclear receptor, FXR and a membrane G protein-coupled receptor, Takeda G protein-coupled receptor 5 (TGR5) in the ileum [17,50,51]. Gut microbiota dysbiosis down regulates the synthesis of these receptors, inducing bacterial translocation and bacterial overgrowth, especially of gram-negative organisms and the perpetuation of gut permeability [12], contributing to increases in LPS, which might activate the nuclear factor kappa-light-chain-enhancer of activated B cells (NFκB) [52] through toll-like receptors (TLRs) and nod-like receptors (NLRs). All of this leads to the production of inflammatory cytokines and chemokines that enter the portal circulation, resulting in inflammation and liver disease progression [53,54]. The involvement of NFKβ in hepatic I/R is well known [55]. Moreover, some secondary BAs produced by the microbiota as deoxycholic acid (DCA) and lithocholic acid (LCA) may regulate the immune system by binding to TGR5 [56]. These secondary BAs may ultimately inhibit NFκB production, thus reducing inflammatory pathways [57]. As it has been mentioned before, dysbiosis causes an alteration in the levels of secondary BAs among which we find DCA and LCA, which could induce inflammation and negatively affect the immune system.

Due to the protective role that FXR and TGR5 exert on liver disease progression, their activation has been proposed as a therapy for liver diseases, with positive results reported in the treatment of NASH [37,58]. More recently, FXR activation via FGF15 has been identified as a way to improve outcomes after brain-dead (BD) donor LT of steatotic and non-steatotic grafts in rats and indeed, BD induced intestinal damage and down-regulation of FXR and the resulting reduction in intestinal FGF15 was associated with low hepatic FGF15 levels, liver damage and regenerative failure. FGF15 administration to BD donors increased hepatic fibroblast growth factor receptor-4 and its co-receptor klotho-beta (FGFR4-KLB), reduced the cytochrome P450 7A1 (CYP7A1) enzyme and normalized BA levels. This was associated with protection against the intestinal damage induced by BD [59]. In steatotic grafts from donation after circulatory death (DCD) donors, FGF15 is not involved in damage or proliferation whereas it protected against BA accumulation, damage and regenerative failure in non-steatotic LT from DCDs [60]. Further studies will be required to determine whether the deficiencies in FGF15 generated by the intestine might induce changes in microbiota or related products and what the potential effects of such changes on the liver and post-operative outcomes after LT are. Nevertheless, this possibility should not be discarded. Indeed, some new advances have shown a relationship between FXR, microbiota and TGR5. The activation of FXR signaling by the FXR agonist, fexarmine (FEX) promoted the increase in LCA produced by the gut bacteria. As LCA is a natural agonist of TGR5, amounts in LCA resulted in the activation of the TGR5 signaling pathway to improve hepatic metabolism [61,62].

In line with this, in preclinical models of steatotic and non-steatotic LT from DCD, it has been observed that alterations in hepatic glucagon-like peptide (GLP-1), which is derived from the intestine, negatively affected post-operative outcomes [63]. The possibility that such alterations might induce changes in the microbiota should not be dismissed. Indeed, it has been indicated that GLP-1 can modulate the gut microbiota composition in both simple obese and diabetic obese subjects [64]. In addition, another preclinical study indicates that an SCFA such as propionate induces GLP-1 synthesis [65]. Thus, given that the microbiota metabolizes SCFAs (which regulate GLP-1 synthesis), it is expected that a dysregulation of the microbiome might negatively affect SCFA and consequently GLP-1 in LT, which has not been reported, to date.

It is well known that in physiological conditions, commensal bacteria are able to produce metabolites such as SCFAs (especially butyrate), which are essential for the health of enterocytes and thus for the permeability of the gut barrier [12]. Although different reports indicate that SCFA exert pro-inflammatory effects and active immune system [66,67], this does not seem to be the case in liver transplantation. Indeed, previous clinical studies in liver transplantation indicate that SCFAs reduce hepatic ischemia-reperfusion injury without adverse effects on the immune system [12,68]. This is in line with different reports indicating that SCFA have anti-inflammatory and immunosuppressive functions [12,68,69,70]. Thus, SCFAs have been shown to reduce NFκB gene expression, ameliorating macrophage activation and to mediate systemic adaptive immune responses by inhibiting cytokine production, and the activation of T cells, while activating regulatory T cells (Treg) [12,52]. It is well known that in liver transplant recipients, the reduction in inflammatory response is required to reduce the hepatic ischemia-reperfusion injury and that immune response needs to be suppressed to protect the transplanted liver from rejection [71]. Therefore, in addition to the problems associated with the alterations in the microbiota itself, microbiota dysregulation may cause alterations in the production of SCFAs [72], affecting the immune system and increasing its activity, which might result in inflammation and a heightened risk of graft rejection.

As it has been explained above, amino acids have an important role in gut–liver axis. In the case of LT, the amino acid glutamine (Gln) has been described as a potential protector of the intestinal barrier permeability and the immune system function. Gln induces enterocyte proliferation, inhibits bacterial translocation to the liver and exerts anti-inflammatory and anti-apoptotic effects [73,74,75]. Similar effects have been described with the use of branched-chain amino acids (BCAAs), which may help to regulate the immune system in LT [76,77]. Moreover, it has been suggested that the amino acids pool may modify the diversity of the gut microbiome [78] but further clinical and preclinical studies will be required to characterize the types of bacteria affected by such amino acids.

Bacterial infections are remarkably prevalent in liver-transplant recipients (LTRs). According to two clinical studies performed by Echaniz et al. (*n* = 152) and Losada et al. (*n* = 149), approximately 70% of LTRs present with post-operative infections, between 34% and 50% of which are bacterial [79,80]. According to other studies, infections caused by gram-negative bacteria are the most common after LT [77], promoting inflammation due to their LPS production ability.

As mentioned above, the role of the microbiota in the gut–liver axis after LT has not yet been fully elucidated. Indeed, some recent clinical studies show no significant microbiota differences in the early post-transplantation stage (seven days post-LT) nor in recipients with acute-cell rejection (ACR) or post-LT bloodstream infections [81,82]. On the other hand, many other clinical and preclinical studies differ from these data, supporting the idea that ischemia-reperfusion injury associated with liver surgery may induce changes in the microbiota, affecting inflammation or liver regeneration [4]. Moreover, some data indicate that the gut barrier can be compromised during LT, affecting gut microbiota. The results from these authors indicate that the microbiota is also modified in ACR. The results of some preclinical (Table 1) and clinical studies (Table 2) are shown below.

The results obtained in preclinical models of LT (none featuring BD or DCD conditions) [83,84,85,86], indicate that the pathological conditions of recipients (for example, cirrhosis) might increase the total numbers of bacteria, and endotoxins, bacterial translocation (BT) and alterations in some markers of intestinal damage, including mucins or TLRs. However, no apparent alterations in the microbiota phyla were observed in recipients, either with normal or cirrhotic livers.

In both studies comparing results of LT isografts and allografts [84,85], endotoxemia, BT, and both intestinal and hepatic damage were all more evident in allografts than in isografts, along with increased *Bacteroides*. On the other hand, differences in the alterations, regarding the type of bacteria, were observed in both studies. This was the case with, for example, *Lactobacillus* and *Bacteroides*. In our view, this might be explained by differences in the duration of the anhepatic phase. Indeed, the ischemia times were similar in both studies (1 h and 48–50 min in Xie [84] and Ren [85], respectively). In addition, neither immunosuppressive drugs nor antibiotics were used in either study. However, the differences between the studies might be explained at least partially by differences in the anhepatic phase duration (15–30 min and 25 min in Xie [84] and Ren [85], respectively). This proposition is supported by the relevance of intestinal congestions occurring during the anhepatic phase [87], which might induce changes in the gut microbiota depending on the damaging effects on the intestine, which, in turn, depend on the phase duration.

Other studies looking at the administration of either antibiotics or probiotics in allograft LT indicate that the number of *Lactobacillus* and *C. leptum* and *Bifidobacterium* spp was greater in the probiotic group than in the antibiotic and allograft groups. By histological analysis, necrosis and inflammation were lower in the antibiotic group than in the allograft group. However, in relation to the hepatic damage, only AST was reduced in probiotic and antibiotic groups as compared with the allograft group [86].

As mentioned above, all results are derived from LT performed in the absence of BD or DCD, which is to say, in very different surgical conditions than those occurring in clinical practice, it should be borne in mind that BD or DCD induction might induce changes in the number and type of microbial phyla as well as in their products. BD and DCD induce important hemodynamic changes, warm hepatic ischemia, hypoperfusion in the mesenteric microcirculation and reduced hepatic blood flow [88,89,90], resulting in important alterations of the mediators generated in the intestine, intestinal damage, dysregulation in BAs and inflammation [59]. All of this negatively affects liver grafts. In addition, given the results shown in the table, all liver grafts were derived from healthy rats. It follows that, in the studies by Xie and Ren, the liver grafts were not subjected to cold ischemia, whereas it is well known that 6–8 h of cold ischemia is commonly used in clinical practice [91]. Therefore, much needs to be done before experimental results can be transferred into clinical practice. Indeed, we still do not know precisely how steatosis (type, grade and so on) affects the gut microbiota, post-operative results or graft survival. In addition, the success or dysfunction of liver grafts may be affected by other factors including donor age [92], cold ischemia times [93], the length of intensive care stays [94] and the type of donor (whether BD or DCD) [88,89]. The effect of such conditions on gut microbiota remains to be elucidated. All these limitations demand that new preclinical studies in the field of LT must be performed in experimental models as close as possible to real clinical conditions (i.e., cadaveric and living donors, proper ischemia times, presence of steatosis, different ages of donors, and different types of death). The effectiveness of a given strategy could differ depending on any of the above surgical conditions. Consequently, protective strategies that work under some conditions may be ineffective or even deleterious when those conditions change.

According to the different clinical studies shown in the table, LT patients have dysbiosis during the initial post-surgery period [15,42,72,81,95,96,97]. Nevertheless, the data indicate that most bacteria are completely recovered after one month, which suggests that pre-transplant gut microbiota changes in patients with cirrhosis or end-stage liver disease may have a more powerful influence on dysbiosis than the LT itself. This suggestion could be supported by the data obtained by Xie et al. (2011) [83], who observed that changes in the microbiota balance were higher in rats with cirrhosis and recipients of cirrhotic liver than in rats with no pre-existing pathology. However, in our view, knowledge of the status of gut microbiota in the patient immediately before implantation of the liver graft and comparison with post-LT analysis will be required before it can be concluded that the surgery itself does not play a key role in alterations in the gut microbiota. According to the studies reported above, some data could be contradictory regarding alterations in the type of bacteria. This might be explained, at least partially, by different immunosuppression regimens as well as by the type and dose of antibiotic used. Indeed, differential effects of immunosuppressive drugs such as tacrolimus or mycophenolate (MPA) on the gut microbiota have been observed [98].

In line with this, mice treated with a combination of different immunosuppressive drugs show an increase of either *E. Coli* pathogenic bacteria or the *Clostridium sensu stricto* genus, modifying gut microbiota balance [98]. Moreover, other preclinical studies report that cyclosporine could ameliorate graft function and avoid graft rejection by decreasing some *Clostridium* clusters and *Enterobacteriaceae* pathogenic bacteria and increasing *Faecalibacterium prausnitzii* beneficial bacteria [99]. Finally, Jiang JW et al. demonstrate that gut microbial disbalance can be due not only to different immunosuppressive agents, but also to the different doses applied. Thus, a middle dose of tacrolimus-FK506 maintains a good graft function, avoids intestinal disruption and maintains a stable microbiota balance. However, this was not the case for a high dose of FK506 since it has been associated with an increase of plasma endotoxin levels [100]. A small clinical study described the relationship between FK506 and microbiota, reporting that the use of FK506 in the short-term increased intestinal permeability in a dose-dependent manner [101]. On the other hand, another clinical study described that the use of FK506 and cyclosporine over 2–3 years have not had an effect on intestinal permeability or endotoxemia [102]. Despite that the studies performed in humans are limited, the data suggest that immunosuppression has an effect on microbiota only in the early post-LT, but further studies are needed to confirm these data.

Similarly, according to results reported in the table, patients received types of antibiotics [15,42,72,81,95,96,97]. Moreover, the antibiotic dose used in transplant recipients varied, which might result in different microbiota populations [103]. In our view, further studies increasing the number of patients will be required. Understanding the effects of dysregulation in the microbiota on the liver grafts and post-operative outcomes remains a challenge. It is well known that LT is conducted in emergency situations and it is very difficult to collect liver samples from the donor, especially in uncontrolled cardiac arrest conditions. In BD conditions, liver donors are often kept in intensive care units for periods of no longer than six hours after diagnosis of BD [104]. The time frame between declaration of brain death and organ procurement provides a small window for obtaining biological samples from the donor in order to analyze gut microbiota and derived products. Rapid and appropriate analysis of such aspects might improve the quality of liver grafts before implantation and consequently post-operative outcomes. This contention is based on previous preclinical studies that indicate that the levels of different mediators, including FGF15 and GLP-1 (both derived from the intestine), were altered in donors after BD or cardiocirculatory death (CD) induction. This resulted in hepatic damage and regenerative failure. On the other hand, the regulation of FGF15 and GLP-1 in donors improved the quality of liver grafts, and consequently post-operative outcomes, including the survival rate of recipients [59,60,63].

Previous studies suggest that, among numerous injurious effects, intestinal microbiota regulate liver tumorigenesis or inflammatory reactions by altering the activity of pro-inflammatory microorganism-associated molecular patterns, bacterial metabolites, natural killer T (NKT) cell-mediated bile acid metabolism and prostaglandin-E2 (PGE2) mediated suppression of antitumor [105]. As a future perspective, randomized clinical studies and studies that include a sufficient number of marginal donor types will be required to elucidate the effect of surgery on the gut microbiota and how it affects the potential alterations in post-operative outcomes. Only armed with these understandings will we be able to solve the current clinical problem of LT, selecting the appropriate strategy to regulate the gut microbiota and minimize potential negative consequences and transferring experimental results into clinical practice.

A recent study aimed at investigating the fecal microbiome of liver recipients with abnormal/normal liver function indicated a decrease of fecal microbiome diversity in recipients with healthy livers and a higher abundance of opportunistic pathogens such as *Klebsiella* and *Escherichia/Shigella* in all liver recipients. The authors established a fecal microbiome index (with specific alterations in *Staphylococcus* and *Prevotella*) that could be used to distinguish between these types of LT [42]. This is of clinical interest, given the relevance of identifying and validating prognostic factors in LT.

## 3. Relationship between Partial Hepatectomy and Gut Microbiota

Since the last century, many investigations have been performed in order to understand the components related to liver regeneration (LR) [106]. Many pathways related to growth factors and cytokines have been investigated [107], such as FGF15, hepatocyte growth factor (HGF) or GLP-1 [108]. It is also known that some disease-related states like steatosis are related to a decrease in liver regeneration capability [109] and exacerbated hepatic I/R injury [110]. When a PH is performed, understanding of LR is required to improve post-operative outcomes and facilitate recovery of a fully functional liver. While mechanisms associated with liver regeneration have been widely researched, not many studies have considered the role of I/R, which is a procedure commonly used in clinical practice to prevent the bleeding during parenchyma resection [4,54,111]. Although the gut microbiota has attracted interest in recent years in different pathologies, few studies have evaluated the role of gut microbiota in post-operative outcomes in hepatic resections [13]. This possibility should not be overlooked, given the relationship between the intestine and the liver, the intestinal congestion occurring during total warm ischemia (which might modify gut microbiota) [112], and the results reporting that even in experimental models of partial hepatic I/R, increases in P-selectin, neutrophil and damage to the intestine were observed [9]. No evaluation of whether these alterations are associated with changes in the gut microbiota or its derived products has been carried out.

Among microbial-related components related to liver recovery after PH, some of them, including endotoxins, BAs, SCFAs and probiotics have been evaluated by different authors [13]. In contrast to LT, the occurrence of the gut metabolite endotoxin is needed for liver regeneration. Indeed, the administration of gut-derived endotoxin induces the release of some hepatotrophic factors and the synthesis of DNA [113], critical for liver regeneration in PH conditions [14]. In hepatic resection, the amount of BA increases rapidly and may impair LR [114]. Also, in many diseases where dysbiosis has been described, such as NAFLD, NASH or cirrhosis, the reduced bacterial conversion of primary BAs to secondary BAs, affecting its regulation and thus probably affecting regeneration [12]. Whether alterations in the gut microbiota might affect the generation of secondary BAs by bacteria, thus affecting liver regeneration in hepatic resections remains to be elucidated. SCFAs are an important component of gut microbial fermentation and their regulation has been associated with many liver diseases [115]. Since they are related to providing energy to both gut and liver [116], their regulation is expected to be important in the first stages of liver regeneration.

BCAAs possess an aliphatic side-chain with a branch. BCAAs promote protein synthesis and glucose metabolism and are involved in fatty acid oxidation. BCAAs favor liver regeneration, nutrition status, and hepatic encephalopathy. BCAAs can also reduce oxidative stress and liver inflammation as it is also involved in lactate production [4]. Concerning liver surgery, a protective effect against I/R injury by amino acid supplementation has been demonstrated in experimental and several clinical studies [117]. For instance, in a study aimed to evaluate the impact of a personalized nutritional protocol with diet and oral BCAA supplementation to 1960 patients that underwent liver resections, authors concluded that intervention was a safe and effective approach that may impact on reducing the length of hospital stay [118]. In the same line, another study suggests that perioperative supplementation of a BCAAs-enriched nutrient-mixture is beneficial in reducing the morbidity associated with and in shortening the duration of hospitalization of patients with chronic liver disease who undergo liver resection for HCC [119]. The gut microbiota and amino acid alterations are involved in the progression of obesity and type 2 diabetes mellitus [120] and changes in gut microbiota and amino acid levels have been reported after surgery [121,122,123]. However, these results [121,122,123] are mainly reported on bariatric surgery, not in hepatic surgical conditions. Moreover, there are few studies indicating the beneficial effects of microbial-related compounds such as glutamine after its supplementation in PH [124,125]. Intensive investigations will be required to elucidate whether the benefits induced by amino acid supplementation are due to a regulation over gut microbiota.

Some bacterial species, like *Bifidobacterium* or *Lactobacillus*, are related to liver injury and regeneration [108]. For example, the composition of *Bifidobacterium* is altered in patients with cirrhosis [126] and a lower quantity of *Bifidobacterium* has been seen in dysbiosis associated with cystic fibrosis [127]. A relationship has also been reported between some *Bifidobacterium* species and elevated interleukin-10 (IL-10) expression [128], associated with hepatoprotective effects [129]. Finally, some studies have aimed to evaluate the effects of the microbiota in liver regeneration and its mechanisms of action. For example, in mice treated with ampicillin, the decrease in ampicillin-sensitive commensal bacteria (*Eubacteria, Lactobacillus* and *Clostridium*) retarded hepatic regeneration after 67% PH (Table 3). This increased interleukin-12 (IL-12) levels, activating hepatic NKT cells, which increased interferon-γ and impeded liver regeneration [130]. In this case, the use of antibiotics might be useful to avoid potential infections, but their effect on the gut microbiota should be considered, since it might negatively affect liver regeneration.

One study, by Xing et al. [112], describes alterations in the microbiota induced by I/R. *Bifidobacteria* and *Lactobacilli* decreased, while *Enterobacterium* and *Enterococcus* increased. This is also related to a decrease of microvilli, damage in the liver, an increase in plasmatic LPS and bacterial translocation to the kidney. Another article related to I/R indicates that supplementation with *Lactobacillus paracasei F19* (LP-F19) restored the alterations in the gut microbiota and this was associated with protection against damage in both steatotic and non-steatotic livers [110].

In two articles [13,14], authors mention dynamic changes in the microbiota and its metabolites during liver regeneration after PH. The microbiota remained altered after the size of the liver was recovered. Also, although both performed a ⅔ PH, their animal species were different (rats and mice), meaning the surgical procedures differed. Moreover, these differences might explain, at least partially, controversial results observed in both studies related to the dynamic profile of microbiota, metabolites and functional pathways described. Indeed, the studies by Liu et al. [13] indicated increases in Bacteroidetes and decreases in *Firmicutes*, whereas in the study reported by Bao et al. [14], the levels of *Bacteroidetes* were decreased and those of *Firmicutes* were increased.

Investigations aimed at evaluating the potential alterations in the microbiota in PH under I/R in livers with pathologies are of scientific and clinical interest since I/R is commonly used in clinical practice to prevent bleeding during resection [6]. This is because this surgical procedure is often performed in subjects that present a disease associated with microbial and metabolite features different from a lean phenotype, such as NAFLD/NASH [131,132] and cirrhosis [20,133]. Studies focused on steatotic livers submitted for surgery might be of scientific and clinical interest since the gut microbiota influences lipogenesis and BAs [134]. Lipogenesis is affected by the absorption of SCFAs in the intestinal lumen by the microbiota [135]. In addition, the microbiota is able to deconjugate BAs and turn them into secondary BAs [136].

It is clear, from all the reported results of the effect of hepatic resections, that the surgical procedure by itself induces changes in the microbiota. However, the effect of these alterations on post-operative outcomes remains to be elucidated. In addition, inconclusive data might be obtained from the literature to date because the studies are limited: there are differing effects on bacterial profile reported for the same surgical procedures and, to our knowledge, no study has been focused on PH under I/R. Although it has been suggested that the products derived from the microbiota might play a key role in hepatic resections, further investigations are required using preclinical models that mimic clinical conditions as closely as possible including the ischemia times as commonly used in clinical practice (e.g., 60 min) and both healthy and pathological livers. In such conditions, SCFAs, endotoxin levels, and secondary BAs should be evaluated. Therefore, depending on the preclinical results, the pharmacological modulation of products derived from the microbiota and consequent effects on liver injury and regeneration should be investigated. All of these factors need to be investigated to develop protection against hepatic damage and regenerative failure using strategies based on microbiota-derived products.

To our knowledge, no clinical studies aimed at evaluating the effects of hepatic resection on potential alterations in the gut microbiota have yet been reported.

## 4. Gut Microbiota-Based Therapy

Herein we show the different strategies reported to date to modulate alterations in the gut microbiota and its derived products.

### 4.1. Pro/Prebiotics

Current evidence has indicated advantages resulting from the use of probiotics to prevent infections after LT without major side effects [137]. Administration of *Bifidobacterium* and *Lactobacillus* ameliorated ischemia-reperfusion injury (IRI) in a mouse model by reducing plasma endotoxin levels and restoring the intestinal barrier function [138]. In addition, in a rat model of LT, such bacteria were increased after the induction of ischemic preconditioning and this reduced IRI [139], which suggest that probiotics effects could be similar to ischemic preconditioning benefits, a surgical technique with a protective role against IRI [91,140]. These effects were all explained by the production of SCFAs, immunomodulators which have the ability to reduce inflammation due to the regulation of macrophage activation [12,68].

A meta-analysis of four controlled studies (*n* = 246) demonstrated that LT patients treated with prebiotics and probiotics before or on the day of transplantation had a reduced infections rate after surgery (7% vs. 35%) and shorter hospital and intensive care unit (ICU) stays as well as a reduction of the duration of antibiotic use [141]. An additional placebo-controlled clinical trial of probiotic treatment on 55 liver-transplant recipients showed significantly reduced 30- and 90-day infection rates, lower post-transplant bilirubin concentration and a more rapid decrease in transaminases [142].

It has been reported that prebiotics might decrease the rate of infections after orthotropic LT because they improve the function of the intestinal barrier, induce the secretion of mucous and immunoglobulin A, improve intestinal motility, decrease the synthesis of inflammatory cytokines and prevent the colonization of the gut by pathogenic bacteria. They can also enhance the production of SCFAs that in turn can be used as energy substrates for the intestinal cells and promote an effect similar to probiotics [143].

The effect of bowel decontamination using gentamycin versus a combination of three probiotics for one week before LT and continued for two weeks after LT on gut microbiota, liver histology, cytokines, and T cells were evaluated. The group treated with probiotics presented lower gut inflammation and a better preservation of gut epithelial barrier. Both antibiotic and probiotic groups showed decreased hepatic injury in the context of ACR. According to these data, the benefits of probiotics were attributed to the induction of intestinal Treg cells, increased levels of transforming growth factor β (TGF-β) in plasma and a decrease in the CD4/CD8 ratio [86,137]. The mechanisms responsible for beneficial effects induced by the antibiotic treatment remain unclear.

On the other hand, according to three controlled trials in which the probiotics formulations contain *Lactobacillus*, prebiotics do not seem to have a clear impact over ACR. While there was a difference in ACR (20% in the probiotic group vs. 28% in the control group), this was not significant [12,141,144,145,146]. Nevertheless, studies that report data about the relationship between ACR and persistent dysbiosis concluded that this dysbiosis (that in part could be reverted thanks to prebiotics and probiotics) may be associated with one year mortality [12].

In I/R, the use of probiotics has also been investigated. For example, it has been reported that the supplementation of *Lactobacillus paracasei F19* reduced the effects of I/R on the liver and on gut microbiota in rats fed with either a standard or steatogenic diet. Of interest, beneficial effects were more evident in the absence of steatosis [110].

### 4.2. Antibiotics

The administration of polymyxin B sulfate (for seven days) in rats undergoing LT reduced *Enterobacteriaceae*, while it increased *Bifidobacterium, Lactobacillus, Bacteroides,* and *Eubacterium*. This was associated with reduced endotoxemia and TNF-α production [147]. Similar effects were reported in one small retrospective study regarding another antibiotic: Rifaximin. It reports the benefits of this antibiotic regarding post-LT infections in patients with end-stage liver disease [148]. Two other retrospective studies conclude the same: that antibiotics such as rifaximin, neomycin, erythromycin and ampicillin-sulbactam prior to LT decreases infection, thus reducing liver injury, inflammation and early allograft dysfunction [4,46,149]. Only one randomized clinical trial assessed the effect of bowel decontamination on gut flora changes by investigating fecal cultures after LT. The study reported less gram-negative bacteria in the bowel decontamination group compared with controls, although no difference in infection rates after LT was noted [150]. Given the data, the role of gut decontamination alone remains unclear and it needs to be clearly elucidated [137]. The possibility of decontamination along with bacterial repopulation is discussed later.

Studies evaluating changes in gut microbial populations and diversity caused by hepatic I/R and their consequences for liver function and regeneration are limited. From 2014 to 2019, authors only examined the effect of therapeutic approaches on intestinal microbiota and hepatic injury and such strategies were mainly based on the use of antibiotics [4]. On the other hand, how the regulation in intestinal microbiota induced by the administration of antibiotics might positively affect hepatic functions remains to be clarified. In addition, none of these studies have been focused on evaluating the effects of the administration of antibiotics on alterations to the microbiota in steatotic or aged livers undergoing surgery.

The administration of antibiotics reduced hepatic injury in LT rats with ACR, but according to this study the gut barrier seems to be altered and disrupted affecting the microbiome diversity [86]. This is of clinical interest because antibiotics might be considered a useful strategy to regulate alterations in the gut microbiota if intestinal integrity is maintained.

As it can be seen above, some data remains unclear and contradictory, so further randomized clinical trials and LT models are required to elucidate the precise mechanisms of action of antibiotics, and how they could affect the liver function throughout the changing of the gut microbiome [4].

It should be noted that although the administration of antibiotics might reduce infections and to improve hepatic injury, other strategies should be evaluated because as shown in previous sections, hepatic injury, alterations in intestinal microbiota and BT, among other effects are present. Moreover, the side effects derived from the administration of antibiotics should be considered. One example is the destruction of the microvilli of the ileum epithelial cells observed in LT, as explained above. The use of antibiotics is associated with multidrug-resistant bacteria. A recent study indicates that multidrug-resistant bacteria colonization could be a major marker of persistent dysbiosis in liver transplant patients, causing this microbiome imbalance to have negative effects on liver function [151].

### 4.3. Diet and Components that Modulate the Gut Microbiota

Some papers have reported that a short-term starvation could ameliorate liver function by reducing necrosis and apoptosis associated with I/R injury [152,153] or PH [154], thus resulting in a better post-operative outcome. However, other papers indicate that starvation could negatively affect I/R associated to either LT or PH [4,155]. In this line, preclinical results in PH indicate depletion of butyrate and alterations in the composition and function of microbiome under overnight fasting conditions that negatively affect the post-operative outcomes [156]. The different effects on starvation depend on the type of the liver submitted to surgery. Thus, the starvation in fatty livers submitted to liver surgery is associated with exacerbated I/R damage [157]. It should also be taken into account several limitations including the time of starvation required in the clinical practice, especially in the case of LT from BD or CD donors by the emergency situation and the derived-logistic problems.

The use of time-restricted feeding (TRF) prior to surgery reduced the damage, oxidative stress and inflammatory biomarkers associated with I/R injury, probably due to intestinal increases in the *Firmicutes* phylum, *Clostridia* and *Bacilli* classes, *Clostridiales* and *Lactobacillales* orders, and *Lachnospiraceae* and *Ruminococcaceae* families, bacteria that are clearly associated with a healthy gut phenotype [4,158,159]. However, different results have been reported regarding the diet restriction since, under these conditions, endotoxemia, increased TNF-α and BT, and less *Bifidobacteria* and *Lactobacillus* in the ileum were observed after LT [160].

On the other hand, it has been reported that the nutritional status previous to the surgery, the damage resulting from LT or PH, along with the type of liver submitted to liver surgery, can increase the risk of post-operative failure [4,76]. Patients with end-stage liver diseases who require LT tend to present malnutrition, as it is also related to microbiota negative features such as dysbiosis and BT [76]. Thus, many groups have reported different diet requirements and compounds to avoid the problems associated with the malnutrition of patients requiring a liver surgery. Enteral immunonutrition and other dietary regimens such as all-trans retinoic acid (RA) seem to have beneficial effects on liver surgeries, promoting liver regeneration. The administration of RA forty-eight hours before PH was associated with a reduced ratio of *Firmicutes* to *Bacteroidetes*, described as a healthy phenotype [109]. Nevertheless, no study clearly identifies a relationship between RA, gut microbiota and liver regeneration, which could lead to the development of new therapeutic strategies. Enteral immunonutrition enriched with nucleotides, arginine, omega-3 fatty acids and hydrolyzed whey peptide (HWP) (started within the first 24 h after surgery) seems to diminish infection risk in liver transplanted recipients, according to one retrospective study [161].

The treatment with glutamine immediately before surgery improves liver regeneration in PH [124] as well as the administration of glutamine during at least three days before LT improves the stability of the intestinal barrier and endotoxemia in LT [162].

In PH and LT, the treatment with probiotics (i.e., *Enterococcus faecalis*, *Clostridium butyricum* or *Bacillus mesentericus*) or synbiotics (the combined administration of prebiotics and probiotics) pre-and post-surgery improves the gut microbiota and reduces the negative effects associated to dysbiosis such as an increase in intestinal permeability, immune alterations and infections, [146,163,164,165].

These are other studies based on the following treatments in liver surgery: lipid emulsion or carbohydrate supplementation just after PH maintain ATP levels, reduces liver damage and increases liver regeneration [166]; the treatment of omega-3 fatty acids three days before liver surgery and one after surgery protects liver against inflammation and damage associated with PH [167]; the treatment with BCAAs prior to surgery improves liver regeneration and decreases liver damage [168,169]; finally, the treatment with dexpanthenol (pro-vitamin B5) immediately before surgery decreases I/R injury [170]. Further investigations will be required to elucidate whether the benefits induced by such compounds in PH and LT are mediated by a regulation in gut microbiota. This possibility should not be discarded because of the numerous papers reported in the literature that describe changes in gut microbiota induced by lipids [171], carbohydrates [172], and amino acids [173] in different liver diseases.

In our view, whether (a) changes in diet regulate dysbiosis and (b) dysbiosis negatively affect the graft viability and the post-operative outcomes in the surgery of hepatic resections and LT, changes in the diet should be applied before surgery to diminish the detrimental effects of PH, BD or CD before livers grafts are retrieved from donors. All of this should be investigated in order to promote better preservation of the organ and to improve the outcome after liver surgery. By the emergency situation of LT, such strategies might be more feasible in the liver surgery of hepatic resections.

## 5. Conclusions

For many years, several therapeutic strategies to reduce damage and regenerative failure in both healthy and pathological livers in resections or transplantation have been attempted, mainly focused on the pathological mechanisms occurring in the liver. However, none have been successfully transferred to clinical practice. In our view, exploration of alternative strategies evaluating not only the liver but also other organs closely related to liver function is required. In particular, the gut, which exhibits a close anatomical and functional bidirectional interaction with the liver, mainly through the portal circulation.

The involvement of alterations in gut metabolites and microbiota in the pathogenesis and progression of NAFLD is well known. However, the role of dysbiosis in the gut microbiota in either LT or PH is yet to be elucidated and current results are controversial. Some clinical studies show no significant microbiota differences in the early post-transplantation stage nor in recipients with acute-cellular rejection or post-LT bloodstream infections, whereas there are many other clinical and preclinical studies with contrary results. Although the gut barrier can be compromised during LT affecting gut microbiota, it is not clear whether pre-transplant gut microbiota in patients with cirrhosis or end-stage liver disease may have a more powerful influence on dysbiosis than LT itself. In our view, comparison of the gut microbiota status in patients (a) immediately before implantation of the liver graft and (b) after LT, would reveal whether the surgery itself plays a key role in the alterations in the gut microbiota observed after LT. To date, preclinical studies focusing on the gut microbiota in LT have not included liver grafts with pathological conditions such as steatotic or aged liver grafts, those with viral infections or grafts from BD or DCD. Future studies that include marginal donors will be required to elucidate the effect of surgery on the gut microbiota when liver grafts exhibit comorbidities that are commonly found in clinical practice. This is extremely important because it is known that the signaling mechanisms in LT can differ greatly between optimal and marginal grafts. In addition, livers with pathological conditions (such as steatosis) exhibit differences in intestinal microbiota diversity in comparison with healthy livers.

Regarding PH conditions, vascular occlusion is a procedure commonly used in clinical practice to prevent bleeding during parenchyma resection. However, very few studies evaluating the role of the gut microbiota in post-operative outcomes in hepatic resections have included I/R in their experimental models. Dynamic changes in microbiota and its metabolites during liver regeneration after PH have been revealed from such investigations. Interestingly, in experimental conditions of I/R without PH, microbiota dysbiosis was also demonstrated after surgical procedures. Taken together, these results seem to indicate that the surgical procedure itself induces changes in the microbiota; however, they cannot be considered conclusive data because the studies are limited and of limited clinical relevance since the vascular occlusion occurring in clinical hepatic resections has not been considered in any such preclinical studies. Thus, future studies that include resection and ischemia to approximate what happens in clinical practice are required. Investigations aimed at evaluating the potential alterations in the microbiota in PH under I/R in pathological livers are also of scientific and clinical interest, because this surgical procedure is often performed in subjects with liver disease (such as NAFLD/NASH or cirrhosis), which is associated with different microbial and metabolite features than those described in healthy livers.

Dysbiosis in the gut microbiota affects signaling pathways such FXR and TGR5, inducing bacterial translocation, among other effects, and contributing to liver disease progression. In addition, dysregulation of intestinal microbiota causes alterations in SCFA generation, increasing immune system activity, which might result in greater risk of graft rejection. As SCFAs are also related to providing energy to the gut and liver, their regulation seems to be important in the early stages of liver regeneration in hepatic resection. In contrast to its occurrence in LT, endotoxin is one of the gut metabolites needed for liver regeneration, which is critical in PH conditions. The role of such metabolites in LT or resection with both healthy and pathological livers remains to be explored. This is especially crucial for amino acids due to the limited studies reported in the literature. Pharmacological modulation of the products derived from the microbiota and their effects on liver injury and regeneration should be investigated to design effective strategies to regulate the gut microbiota and improve post-operative outcomes in liver surgery.

The interrelation between the gut microbiota and liver surgery presents a huge area of opportunity for future therapeutic perspectives. Therapies based on gut microbiota regulation, such as treatments with probiotics, prebiotics, antibiotics, preoperative fasting or pre and post-operative diet have promising results in hepatic I/R injury, gut microbiota and liver regeneration. Further studies, mainly focused on the effects of preoperative fasting or pre and post-operative diet on gut microbiota in liver surgery are required. It should also be considered that the types of liver, surgical procedures as well as the nutritional status of the subjects prior to surgery differentially affect the microbiome. Thus, specific protective strategies to regulate the microbiome alterations and consequently the post-operative outcomes in PH and LT might be required.

The administration of antibiotics should be undertaken with caution, since in rats with acute rejection after LT, it reduced hepatic damage but led to injury to intestinal integrity, thus inducing alterations in the microbiota. Further research is necessary to elucidate the molecular signaling pathways through which antibiotics may exert their actions, whether the protection against hepatic damage induced by antibiotics is exerted through changes in the gut microbiome, and to determine the optimal dose and duration of treatments. Moreover, the side effects of antibiotics, such as the presence of multidrug-resistant bacteria, should be taken into account. Understanding the relationship between the gut microbiota and liver surgery could also generate new biomarkers of liver status in resection or LT. Interesting results from several studies suggest that multidrug-resistant bacteria colonization could be a major marker of persistent dysbiosis and liver function in LT recipients and fecal microbiome diversity might be used to distinguish either abnormal or normal liver function after LT.

Preclinical studies exploring the interrelation of the gut microbiota and liver surgery are notorious for their failure to reflect what actually occurs in clinical practice. Experimental studies have been carried out in the absence of BD or DCD, which means in surgical conditions very different to those occurring in clinical practice. BD and CD induce important hemodynamic changes, hypoperfusion in the mesenteric microcirculation and warm hepatic ischemia, which might result in important alterations in the mediators generated in the intestine, intestinal damage, dysregulation in BAs and inflammation. All of this might induce changes in the diversity of the microbiota as well as in its metabolic products and exert injurious effects on liver grafts submitted for transplantation. In addition, experimental studies of the gut microbiota and LT have not included pathological liver grafts, and in some such studies, liver grafts were not subjected to cold ischemia, whereas it is widely known that 6–8 h of cold ischemia is commonly used in clinical practice. In addition, the success or dysfunction of liver grafts may be affected by other factors including donor age, presence of steatosis, cold ischemia times and type of donor (BD or CD). These conditions should be considered in future research to comprehend how they influence the gut microbiota. Regarding liver resection, there is also a lack of research using experimental models that best mimic clinical conditions, for example, the ischemic times commonly used or including both healthy and pathological livers. Of relevance, post-operative outcomes in liver resection are influenced by the presence of pathologies in liver, such as steatosis or cirrhosis, because they increase hepatic damage and impair regeneration. Because the effectiveness of a therapeutic strategy aimed at regulating potential alterations in the gut microbiota and its derived products could differ depending on surgical conditions, type of liver and other factors, the use of experimental models that reproduce as closely as possible real clinical conditions is especially important to afford an effective translation of laboratory results to patients.

So far, research on the interrelation between the gut microbiota and liver surgery is in its infancy and there is still much to do. The knowledge that has been generated to date is very limited and it still does not allow us to clearly distinguish the alterations that the intestinal microbiota or its metabolic products induce on postsurgical outcomes in LT or resection; nor whether it is the surgery itself that causes such alterations that could in turn affect post-operative results. Much less is known about the molecular mechanisms involved in these events, and therefore, therapeutic targets that can be used in the design of future treatments in liver surgery, based on the regulation of the gut microbiota or its metabolic products have yet to be defined. The discoveries generated from the first investigations into the complex interrelationship between the microbiota and liver surgery clearly show the need for the design of appropriate preclinical models of surgery that closely approximate clinical practice. This is crucial for establishing therapies to combat hepatic damage and regenerative failure based on microbiota regulation, which can be successfully translated into clinical practice.

## Figures and Tables

**Table 1:** Preclinical studies in liver transplantation (LT).

Study	Surgery (DCD/BD)	Recipients Characteristics	Alteration Post-LT
Xie et al. (2011) [83]	OLT without DCD/BD	Normal SD rats (donors) and normal SD or SD with cirrhosis (recipients). Groups: -LN (*n* = 12; 6 transplanted rats) -LTC (*n* = 14; 7 transplanted rats)	↑ endotoxin, BT and bacteria number in LTC rats. = *Lactobacilli, Bacteroides* and *Enterobacteria* between groups. ↑ MUC2/3 and TLR4 in LTC rats. Slight rejection and periportal inflammatory cell infiltration in both LN and LTC rat
Xie et al. (2012) [84]	OLT without DCD/BD	L and BN rats (donors) and BN rats (recipients). Groups: -BN rats transplanted from L rats (*n* =12; 6 transplanted BN rats) -BN rats transplanted from BN rats (*n* = 12; 6 transplanted BN rats)	↑ endotoxin and BT to the liver with AR ↑ *Bacteroides* associated to AR = *Clostridium leptum, Enterobacteriaceae* and *Lactobacillus* after 1–2 weeks post-LT Better hepatic architecture in isograft group ↑ Liver function index in allograft group.
Ren et al. (2014) [85]	OLT without DCD/BD	L and DA rats (donors) and L rats (recipients). Groups: -NR: OLT from L rats to L rats (*n* = 18; 9 transplanted rats) -AR: OLT from DA (specific-pathogen free rats) rats to L rats. (*n* = 18; 9 transplanted rats)	↓ *Faecalibacterium prausnitzii* and *Lactobacillus* during AR ↑ *Clostridium bolteae* during AR Altered intestinal integrity ↑ endotoxin ↑ hepatic injury in AR rats
Xie et al. (2014) [86]	OLT without DCD/BD	L (donors) and BN rats (recipients). Groups: Allograft group (*n* = 16; 8 transplanted rats) Antibiotic group (*n* = 16; 8 transplanted rats) Probiotic group (*n* = 16; 8 transplanted rats)	↓ endotoxemia in the antibiotic ↑ *Lactobacillus*, *C. leptum* and *Bifidobacterium* in the probiotic group = *Bacteroides* in all groups at 7 and 14 days after LT ↓ Necrosis, inflammation and AST in the antibiotic and probiotic groups

↑: Increase; ↓: Decrease; AR: Acute rejection; AST: Aspartate transaminase; BN: Brown Norway; BT: Bacterial Translocation; L: Lewis; LT: Liver transplantation; LTC: Liver transplantation in cirrhotic receptors; LN: Liver transplantation in normal receptors; MUC2: Mucin 2; MUC3: Mucin 3; NR: Non-rejection group; OLT: Orthotropic liver transplant; SD: Sprague Dawley; TLR4: Toll-like receptor 4.

**Table 2:** Clinical studies in LT.

Study	Surgery (DCD/BD)	Population	IS	Antibiotics	Alterations Post-LT
Wu et al. (2012) [72]	No specified	-28 healthy volunteers -51 cirrhotic patients -111 liver-transplanted patients with cancer or cirrhosis.	-GC -MPA -CNIs	No antibiotics use	↓ *Eubacteria*, *Bifidobacterium* spp., *Faecalibacterium* *prausnitzii* and *Lactobacillus* spp. in LTR. ↑ *Enterobacteriaceae* and *Enterococcus* spp. in LTR *Enterococcus* spp. showed a tendency to restore to normal levels ↑ Plasma endotoxin, IL-6 and fecal sIgA in cirrhotic patients but not in LTR
Lu et al. (2013) [95]	No specified	12 OLT recipients with liver failure from hepatitis B virus (HBV) liver cirrhosis	Different for each patient	No antibiotics use	↓ microbial diversity in some patients Presence of infection in some patients
Bajaj et al. (2017) [96]	No specified	45 LT patients with cirrhosis	-Steroids -MPA -Tacrolimus	TMP-SMX	Dysbiosis after LT
Kato et al. (2017) [81]	Deceased and living donor	38 LT patients (exclusion of patients with fulminant hepatitis or with a previous transplant)	-Tacrolimus -MPA -Steroids	13 received broad spectrum of antibiotics	↓ microbiota diversity associated with high ESLD scores and ACR ↑ *Bacteroides*, *Enterobacteriaceae*, *Streptococcaceae* and *Bifidobacteriaceae* ↓ *Enterococcaceae*, *Lactobacillaceae*, *Clostridiaceae*, *Ruminococcaceae* and *Peptostreptococcaceae*
Sun et al. (2017) [97]	No specified	9 LT with ESLD (4 patients with cirrhosis and 5 with HCC) 15 healthy controls	-Tacrolimus -MP	Cephalosporin	↓ *Actinobacillus*, *Escherichia*, and *Shigella* after LT ↑ *Micromonosporaceae*, *Desulfobacterales*, the *Sarcina* genus of *Eubacteriaceae*, and *Akkermansia*
Bajaj et al. (2018) [15]	Deceased donors	-40 patients with cirrhosis -Patients are their own controls	-Steroids -MPA -Tacrolimus	TMP-SMX	↑ bacterial action after LT
Lu et al. (2019) [42]	No specified	-Healthy controls (*n* = 61) -LTR with HCC (*n* = 90): 42 with LTA and 48 with LTN	-FK506 tacrolimus	No antibiotic use in the 12 weeks prior to enrollment	↓ fecal microbiome diversity in recipients in the LTA group ↑ opportunistic pathogens such as *Klebsiella* and *Escherichia/Shigella* in all LTR ↓ beneficial butyrate-producing bacteria in LTR

↑: Increase; ↓: Decrease; ACR: Acute cell rejection; CNIs: Calcineurin Inhibitors; ESLD: End-stage liver disease; GC: Glucocorticoids; HCC: Hepatocellular carcinoma; IL: Interleukin; IS: Immunosuppression; LTA: Abnormal liver function; LTN: Normal liver function; LTR: Liver transplant recipient; MP: Methylprednisolone; MPA: Mycophenolate; sIgA: Secretory IgA; TMP-SMX: Trimethoprim-sulfomethoxazole.

**Table 3:** Preclinical studies in partial hepatectomy (PH).

Study	Surgery (IR/PH)	Population	Treatment	Effect/Alteration Post-Surgery
Xing et al. (2005) [112]	-I/R (20 min ischemia and 22 h of reperfusion)	-SD rats with normal liver. Groups: -Sham -I/R *n* = 6–10/group	No specified	I/R→↑ALT, AST, MDA, LPS and ↓SOD. I/R→↓*Bifidobacteria*, *Lactobacilli* and ↑*Enterobacterium*, *Enterococcus*. I/R→↑BT to kidney, ↓intestinal microvilli.
Nardone et al. (2010) [110]	-I/R (30 min ischemia and 60 min reperfusion)	-Male W rats with healthy or steatotic liver. Groups: -Rats fed with Standard or Steatogenic diet -Rats fed with Standard or steatogenic diet + 8 week probiotic supplementation *n* = 7–10/group	LP-F19 supplementation	I/R→↓*Bacteroides*, *Bifidobacterium* and *Lactobacillus* and ↑*Enterococcus*, and *Enterobacteriaceae* I/R→necrosis, leukocyte infiltration, ↑MDA, TNFα, IL-1β, IL-6, ALT, AST and LPS, (especially in steatotic livers). LP-F19→↓I/R injury in steatogen diet, ↓↓I/R in standard diet.
Wu et al. (2015) [130]	-PH (67%)	-Male C57Bl/6 mice with healthy liver. Groups: -Mice undergoing PH -Mice undergoing PH+Atb treatment: Amp Neo, Met, Vanco (in combination or separately). *n* = 3–6/group	(Atb-treatment): Amp, Neo, Met, Vanco	Atb in combination: no affect hepatic damage (AST and ALT) but ↓hepatic proliferation (↓PCNA, BrdU-positive hepatocytes). Vanco, Neo, Met alone→no effect on LR. Amp→↓Amp-sensitive commensal bacteria (↓many gram-negative and gram-positive bacteria →↑Kupffer cells and ↑IL-12→↑activation NKT cells→↓LR
Liu et al. (2016) [13]	-PH (2/3)	-Male C57BL/6 mice with healthy liver Groups: -Control -Mice undergoing PH Time: 0 h-9 days. *n* = 3, 4/group.	No specified	-PH: ↑*Bacteroidetes* (S24, *Rikenellaceae*), ↓*Firmicutes* (*Clostridiales*, *Lachnospiraceae*, and *Ruminococcaceae*). -*Ruminococus*, *Bifidobacterium*, *Lactobacillus* and *Clostridium* modulate bile acid conversion ↓*Firmicutes/Bacteroidetes* ratio is associated to lean microbiota; ↑Ratio: obese mice. High fat diet: ↑*Rikenellaceae* and ↓*Ruminicoccaceae*. Exercice: ↑S24-7→↑butyrate ↑Ki67 following PH and liver size was restored at 7–9 days.
Bao et al. (2020) [14]	-PH (2/3)	Adult male SD rats (*n* = 126) with healthy liver. Groups: -Control -Rats undergoing PH -Rats undergoing PH+Atb treatment -Rats undergoing PH+Atb +FMT treatment Time: 0–336 h	-Atb-treatment: Amp, Vanco, Metro, Neo (in combination) -FMT treatment	PH: -Inverse relation between *Bacteroidetes* (*Bacteroidaceae*, *Prevotellaceae*, *Rikenellaceae*, *Porphyromonadaceae*) and *Firmicutes* (*Ruminococcaceae*, *Lachnospiraceae*). *Bacteroidetes* ↓12–24 h, ↑30–48 h and ↓3–14 days *Proteobacteria* ↑48 h PH: ↑Ki67, BrdU, TNFα, IL-6 and HGF. -Atb treatment impaired LR (↓Ki67 and BrdU) and FMT treatment restored LR

↑: Increase; ↓: Decrease; Amp: Ampicillin; ALT: Alanine transaminase; AST: Aspartate transaminase; Atb: Antibody; BrdU: Bromodeoxyuridine; BT: Bacterial translocation; FMT: Fecal microbial transplantation; HGF: Hepatocyte growth factor; I/R: Ischemia/reperfusion; Ki67; IL-1β: Interleukin-1 β; IL-6: Interleukin-6; IL-12: Interleukin-12; marker of proliferation Ki-67; LP-F19: Lactobacillus paracasei; LPS: Lipopolysaccharide; LR: Liver regeneration; MDA: Malondialdehyde; Met: Metronidazole; Neo: Neomycin sulfate; NKT: Natural killer T; PH: Partial hepatectomy; PCNA: Proliferating cell nuclear antigen; SD: Sprague Dawley; SOD: Superoxide dismutase; TNFα: Tumor necrosis factor α; Vanco: Vancomycin; W: Wistar.

## Data Availability

Not applicable.

## Abbreviations

AAs	Amino acids
ACR	Acute-cell rejection
ALD	Alcoholic liver disease
ALT	Alanine transaminase
Amp	Ampicillin
AR	Acute rejection
AST	Aspartate transaminase
Atb	Antibody
BA	Bile acid
BCAA	Branched-chain amino acid
BD	Brain-dead
BN	Brown Norway
BrdU	Bromodeoxyuridine
BT	Bacterial translocation
CD	Cardiac death
CNIs	Calcineurin inhibitors
CVD	Cardiovascular disease
CYP7A1	Cytochrome P450 7A1
DCA	Deoxycholic acid
DCD	Donation after circulatory death
ESLD	End-stage liver disease
FGF15	Fibroblast growth factor 15
FGFR4-KLB	Fibroblast growth factor receptor-4 and its co-receptor klotho-beta
FMT	Fecal microbial transplantation
FXR	Farnesoid X receptor
GC	Glucocorticoids
GLP-1	Glucagon-like peptide 1
GM	Gut microbiota
Gln	Glutamine
HCC	Hepatocellular carcinoma
HGF	Hepatocyte growth factor
HWP	Hydrolyzed whey peptide
I/R	Ischemia/reperfusion
ICAM	Intercellular adhesion molecule
ICU	Intensive care unit
IL	Interleukin
IL-1β	Interleukin-1 β
IL-6	Interleukin-6
IL-10	Interleukin-10
IL-12	Interleukin-12
IRI	Ischemia/reperfusion injury
IS	Immunosuppression
Ki67	Marker of proliferation Ki-67
L	Lewis
LCA	Lithocholic acid
LD	Linear dichroism
LN	Liver transplantation in normal receptors
LP-F19	Lactobacillus paracasei F19
LPS	Lipopolysaccharides
LR	Liver regeneration
LT	Liver transplantation
LTA	Abnormal liver function
LTC	Liver transplantation in cirrhotic receptors
LTN	Liver transplantation in normal receptors
LTN	Normal liver function
LTRs	Liver-transplant recipients
MDA	Malondialdehyde
Met	Metronidazole
MP	Methylprednisolone
MPA	Mycophenolate
Muc2	Mucin 2
Muc3	Mucin 3
NAFLD	Non-alcoholic fatty liver disease
NASH	Non-alcoholic steatohepatitis
Neo	Neomycin sulfate
NFκB	Nuclear factor kappa-light-chain-enhancer of activated B cells
NKT cell	Natural killer-T cell
NLRs	Nod-like receptors
NR	Non-rejection group
OLT	Orthotropic liver transplant
PCNA	Proliferating cell nuclear antigen
PGE2	Prostaglandin E2
PH	Partial hepatectomy
RA	All-trans retinoic acid
ROS	Reactive oxygen species
R TGR5	Receptor of Takeda G protein-coupled receptor 5
SCFAs	Short-chain fatty acids
SD	Sprague Dawley
sIgA	Secretory IgA
SOD	Superoxide dismutase
TGF-β	Transforming growth factor β
TGR5	Takeda G protein-coupled receptor 5
TJ	Tight junction
TLR	Toll-like receptor
TLR4	Toll-like receptor 4
TMP-SMX	Trimethoprim-sulfamethoxazole
TNF	Tumor necrosis factor
TNFα	Tumor necrosis factor α
Treg	Regulatory T cells
TRF	Time-restricted feeding
Vanco	Vancomycin
W	Wistar
